# Supplementary material for: Structure of Trypanosoma peroxisomal import complex unveils conformational heterogeneity
Source: Nat Commun. 2025 Dec 11;16:11398. doi: 10.1038/s41467-025-66207-8 (PMC12738837; doi:10.1038/s41467-025-66207-8)
Supplement: Supplementary file 1 — Supplementary Information [file 41467_2025_66207_MOESM1_ESM.pdf]

Supplementary Material

Structure of Trypanosoma peroxisomal import complex unveils conformational heterogeneity

Ravi R. Sonani[1,&], Artur Blat[1,2*], Malgorzata Jemiola-Rzeminska[3,*], Oskar Lipinski[1,2,$,*], Stuti N. Patel[1,*], Tabassum Sood[1,2,*], Grzegorz Dubin[1,#]

[1] Malopolska Centre of Biotechnology, Jagiellonian University, Krakow, Poland

[2] Doctoral School of Exact and Natural Sciences, Jagiellonian University, Krakow, Poland

[3] Department of Plant Physiology and Biochemistry, Faculty of Biochemistry, Biophysics and Biotechnology, Jagiellonian University, Krakow, Poland

[&] Current address: Department of Biochemistry and Molecular Genetics, University of Virginia School of Medicine, Charlottesville, VA 22903, USA

[$] Current Address: Universite Claude Bernard Lyon 1, CNRS, Tissue Biology and Therapeutic Engineering Laboratory (LBTI), UMR 5305, F-69367 Lyon, France

[*]Equal contribution, listed alphabetically

[#] correspondence to: grzegorz.dubin@uj.edu.pl

This Supplementary Material file contains 18 supplementary figures, four supplementary tables, and supplementary methods.

Supplementary Figure 1

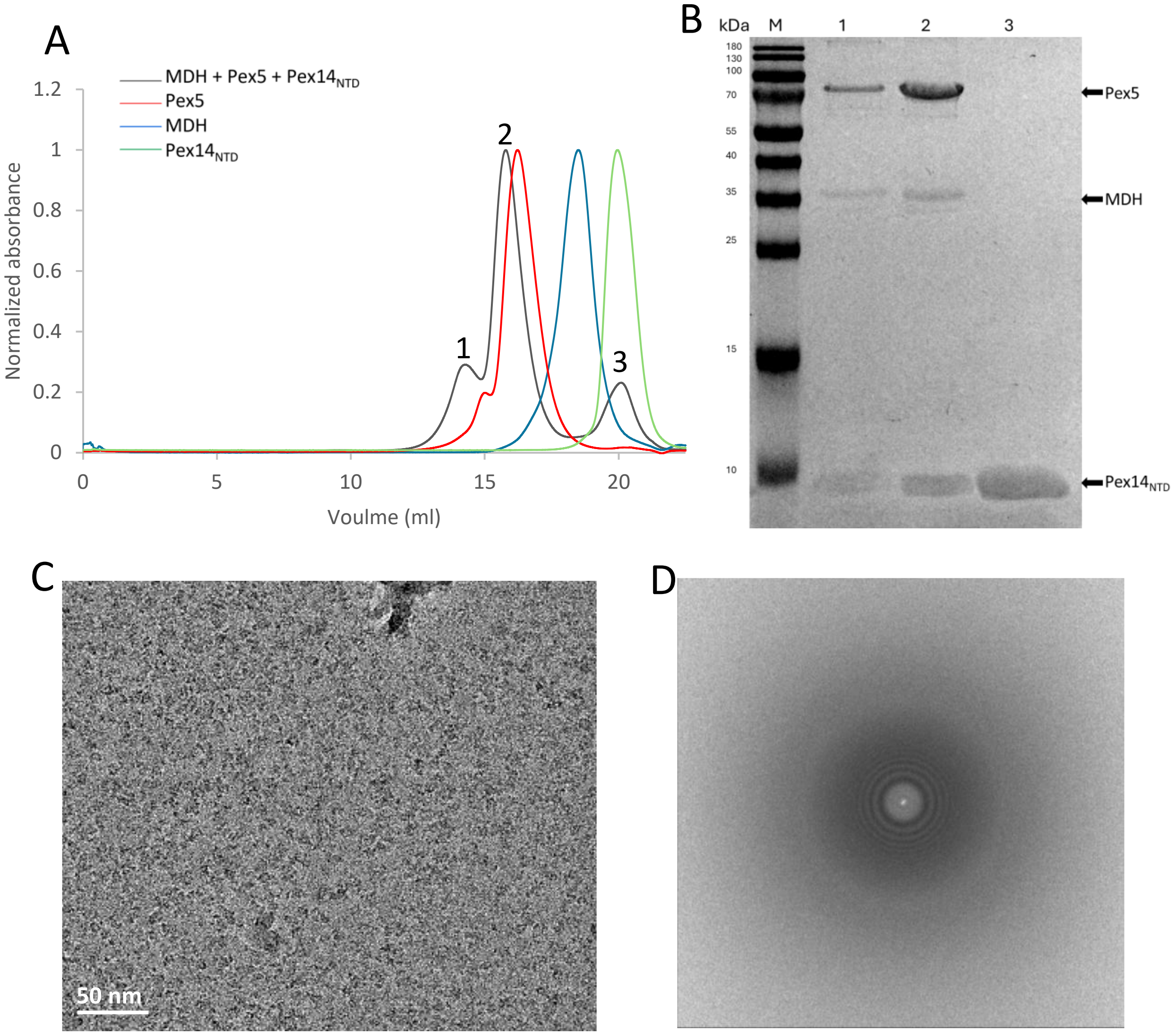

Supplementary Figure 1: (**A**) Size exclusion chromatography (SEC; Superose6 10/300) profile of *in vitro* reconstituted ternary complex MDH-Pex5-Pex14$_{NTD}$ from *T. cruzi*. MDH, malate dehydrogenase. **(B)** SDS-PAGE of the peak 1, 2 and 3 of SEC elution shown in **A**. **(C)** Representative motion-corrected cryo-EM micrograph (out of total 8169 micrographs) of ternary complex MDH-Pex5-Pex14$_{NTD}$. Defocus range for the whole dataset: -3.0 to -0.9 µm. **(D)** Power spectrum of the cryo-EM micrograph shown in **C**.

Supplementary Figure 2

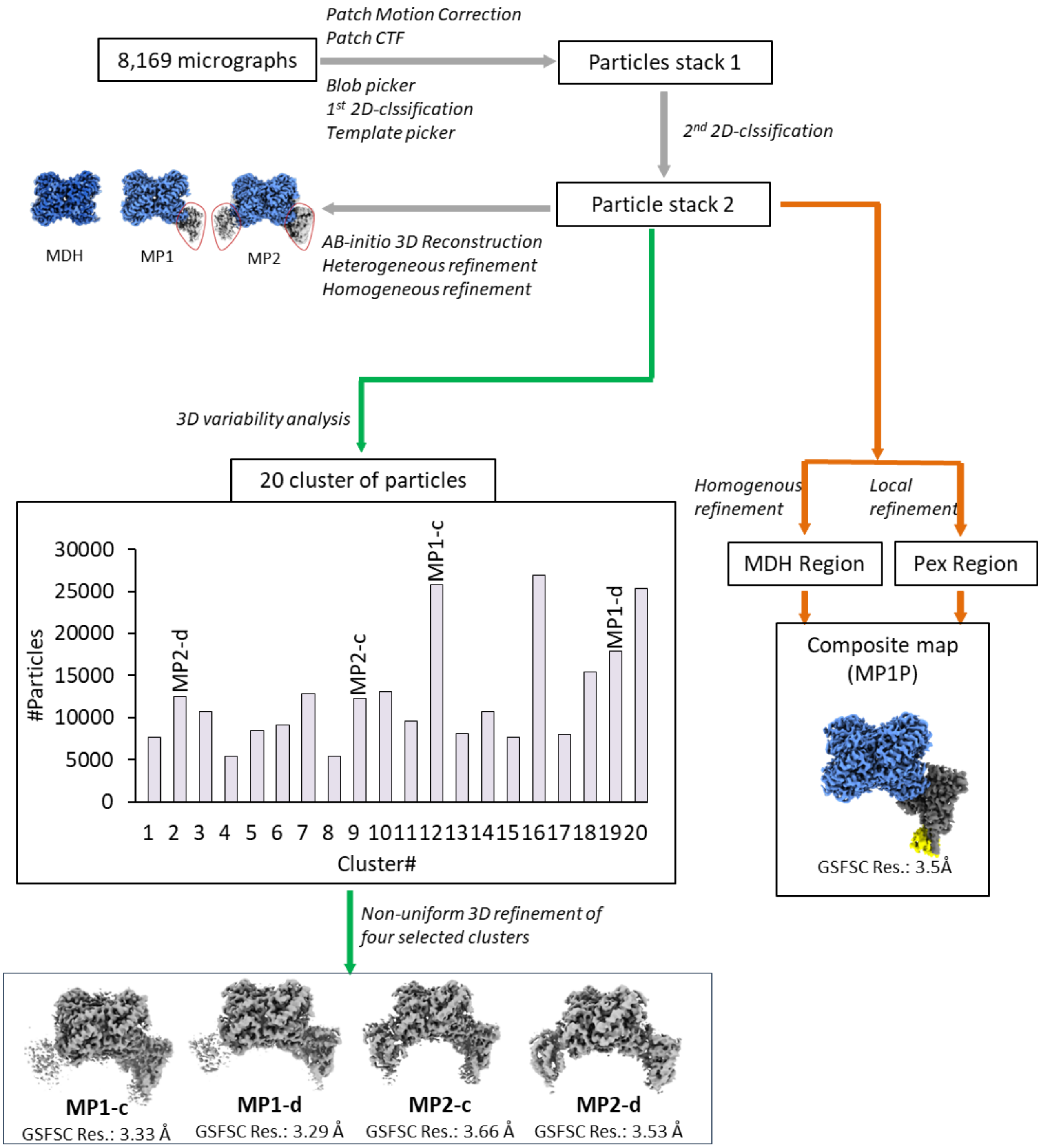

Supplementary Figure 2: Cryo-EM data processing workflow used to derive maps of *T. cruzi* MP1-c. MP1-d, MP2-c, MP2-d and MP1P complexes.

Supplementary Figure 3

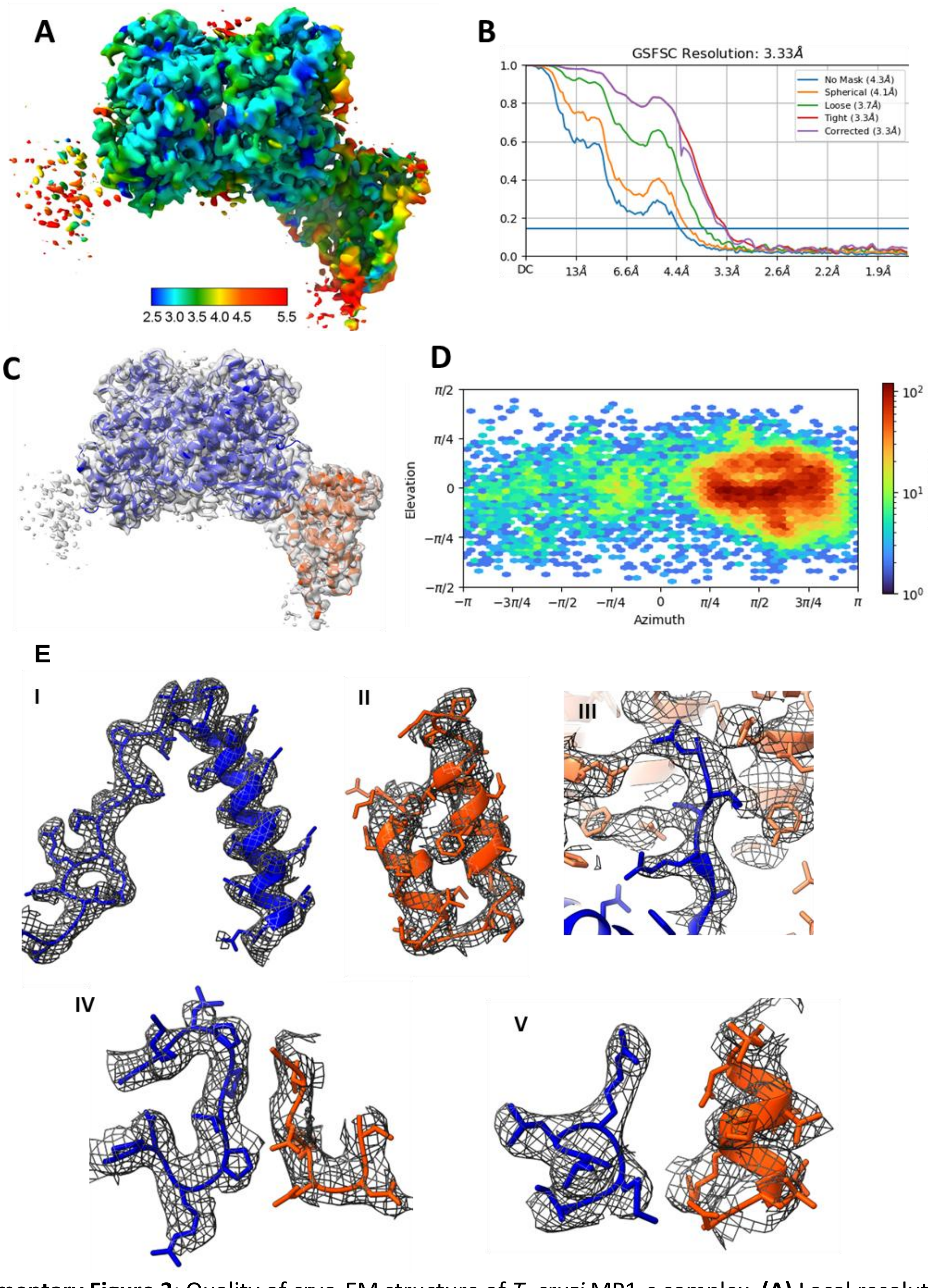

Supplementary Figure 3: Quality of cryo-EM structure of *T. cruzi* MP1-c complex. **(A)** Local resolution map, **(B)** Half maps Fourier Shell Correlation (FSC, threshold 0.143) curve, **(C)** Overall fit of model into density map, **(D)** Angular distribution of particles, and **(E)** Model-vs-density fit in representative regions of MDH (I), Pex5 (II), PTS1 (III), MDH-Pex5 interactions (IV and V). MDH and Pex5 are shown in blue and orange ribbon models, respectively.

Supplementary Figure 4

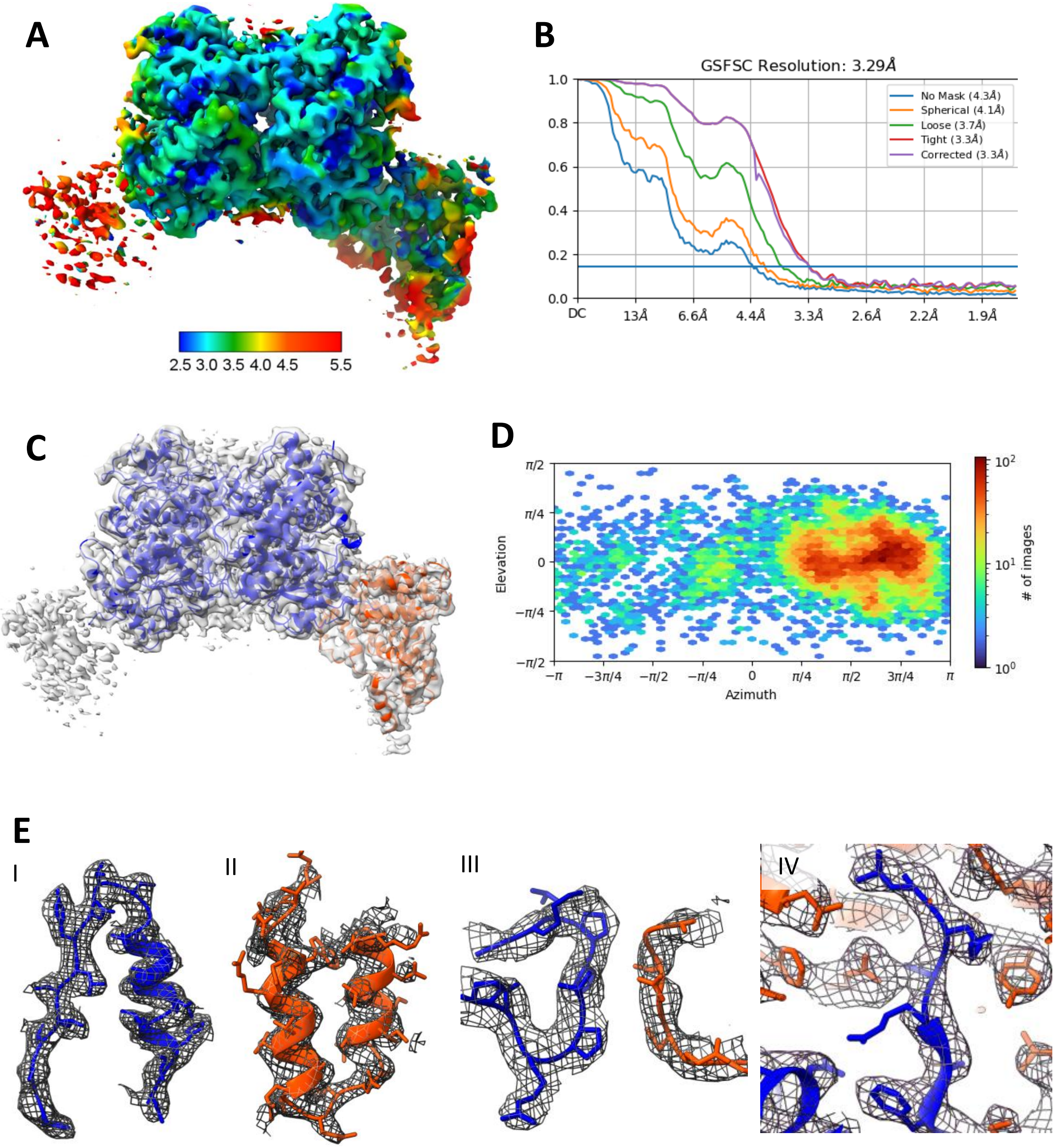

Supplementary Figure 4: Quality of cryo-EM structure of *T. cruzi* MP1-d complex. **(A)** Local resolution map, **(B)** Half maps Fourier Shell Correlation (FSC, threshold 0.143) curve, **(C)** Overall fit of model into density map, **(D)** Angular distribution of particles, **(E)** Model-vs-density fit in representative regions of MDH (I), Pex5 (II), MDH-Pex5 interaction (III) and PTS1 (IV). MDH and Pex5 are shown in blue and orange ribbon models, respectively.

Supplementary Figure 5

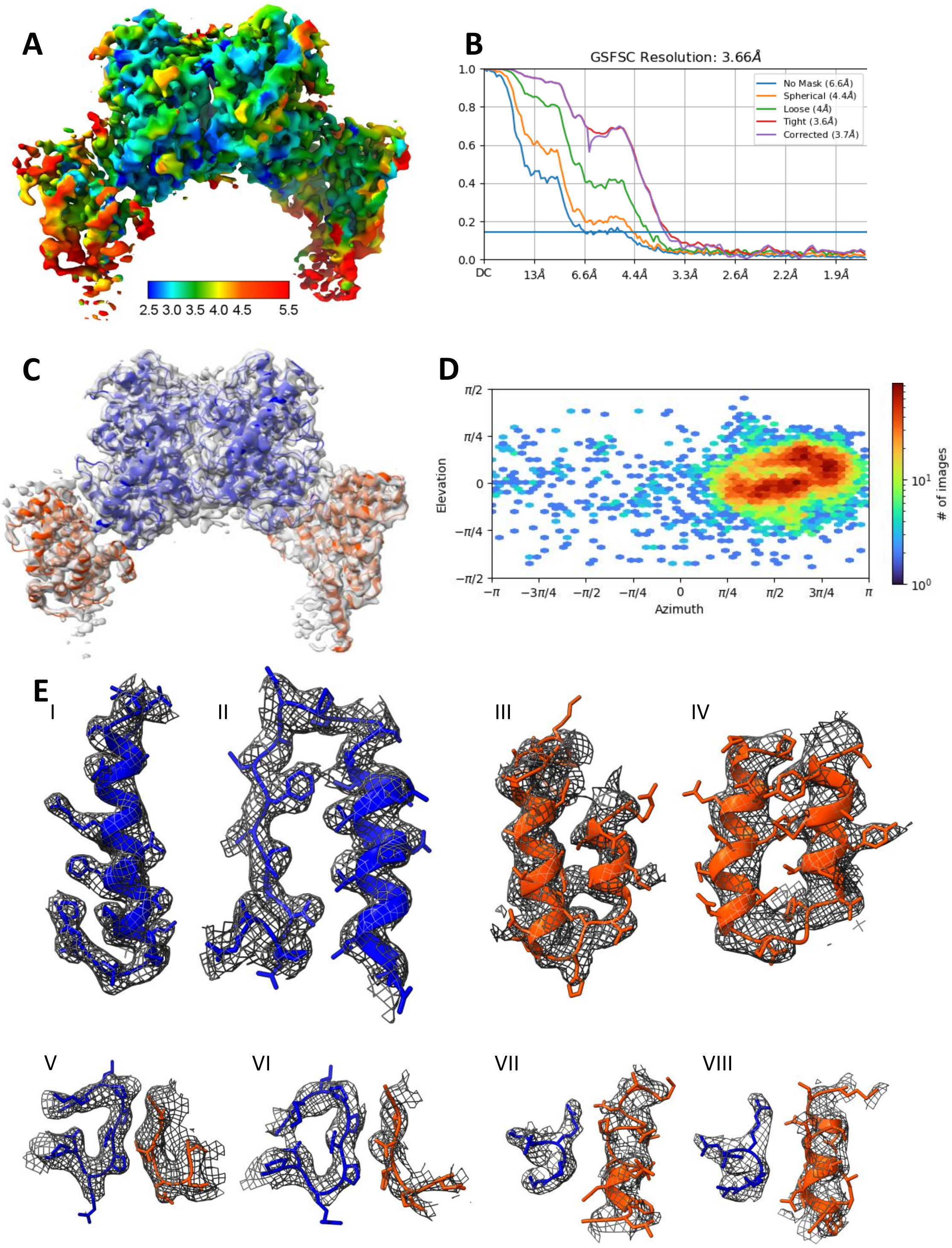

Supplementary Figure 5: Quality of cryo-EM structure of *T. cruzi* MP2-c complex. **(A)** Local resolution map, **(B)** Half maps Fourier Shell Correlation (FSC, threshold 0.143) curve, **(C)** Overall fit of model into density map, **(D)** Angular distribution of particles, **(E)** Model-vs-density fit in representative regions of MDH (I, II), Pex5 (III, IV), MDH-Pex5 interaction (V-VIII). MDH and Pex5 are shown in blue and orange ribbon models, respectively.

Supplementary Figure 6

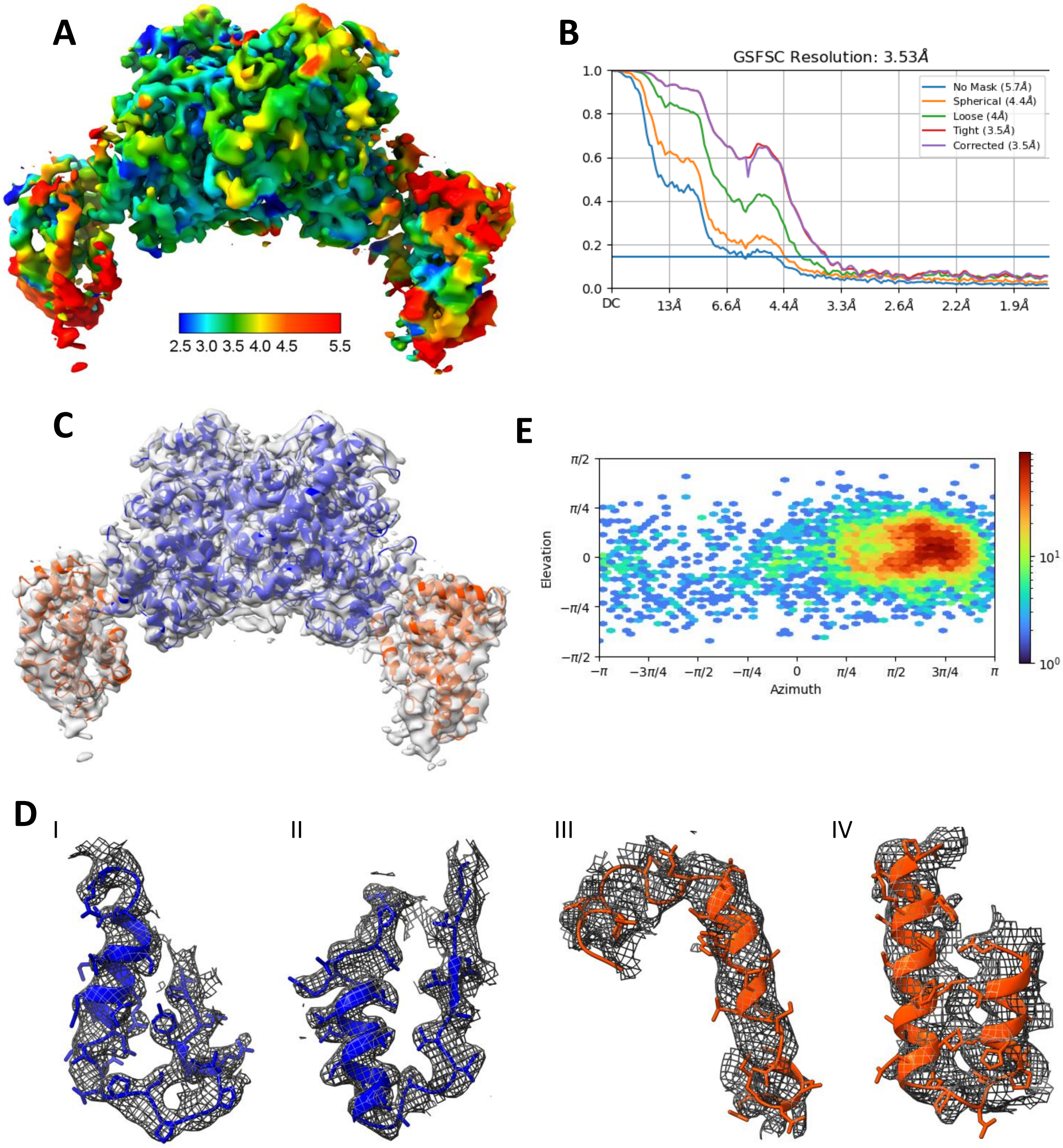

Supplementary Figure 6: Quality of cryo-EM structure of *T. cruzi* MP2-d complex. **(A)** Local resolution map, **(B)** Half maps Fourier Shell Correlation (FSC, threshold 0.143) curve, **(C)** Overall fit of model into density map, **(D)** Angular distribution of particles, **(E)** Model-vs-density fit in representative regions of MDH (I, II) and Pex5 (III, IV). MDH and Pex5 are shown in blue and orange-ribbon models, respectively.

Supplementary Figure 7

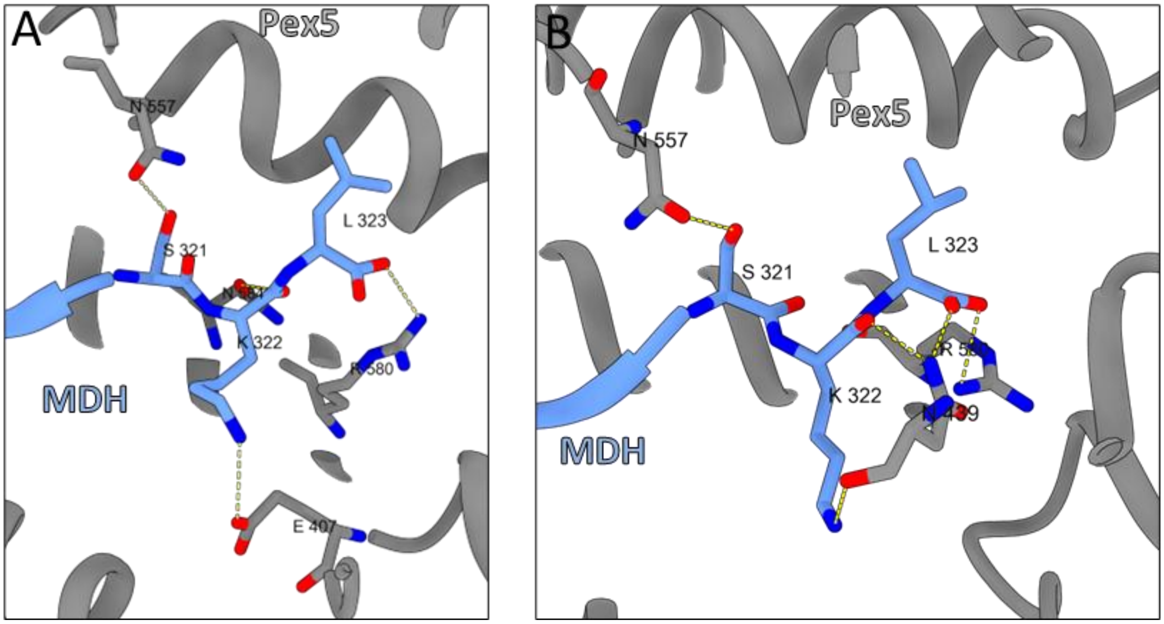

Supplementary Figure 7: Hydrogen bonding network at MDH PTS1 and Pex5 interface in **(A)** distal and **(B)** close states (for definition of states see manuscript).

Supplementary Figure 8

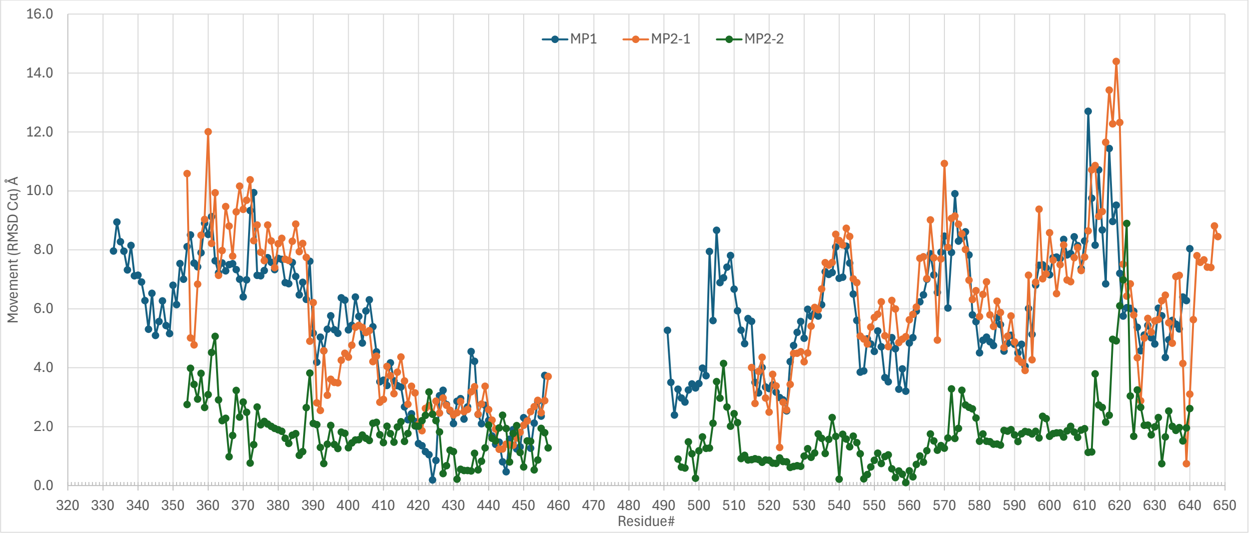

Supplementary Figure 8: Residue wise RMSD of Pex5 relative to the MDH in MP1 and MP2 complexes. The distal and close models were superimposed using MDH structure and the RMSD for Cα of each residue of Pex5 was determined in ChimeraX[1]. MP2-1 and MP2-2 denotes the two copies of Pex5 bound to MDH in MP2 structure. Gaps in the plot indicate the missing residues in the compared model(s).

Supplementary Figure 9

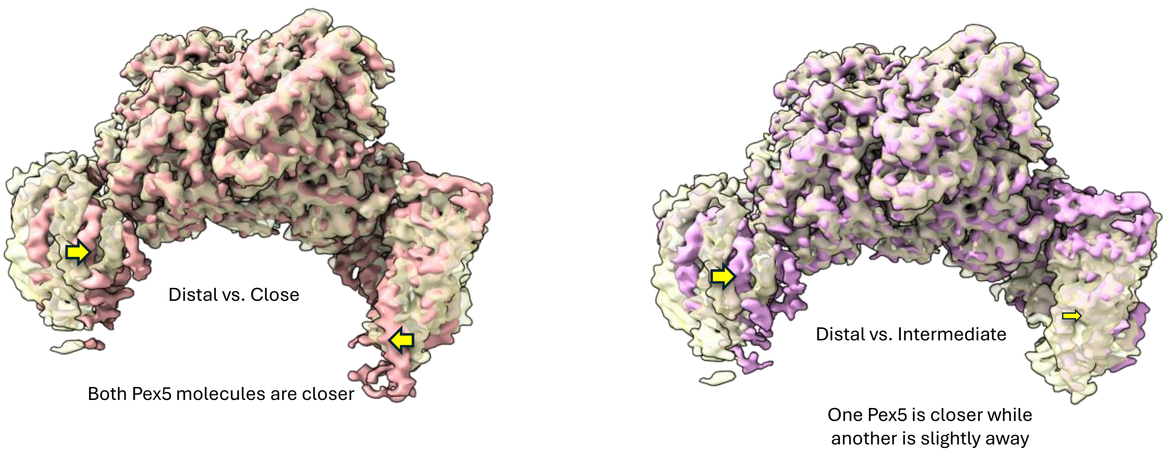

Supplementary Figure 9. **Orientations of two Pex5 molecules are not coordinated in MP2 complex.** (Left panel) Overlay of 3DVA derived densities representing distal (gold; largest opening angle of each Pex5 molecule on MDH) and close (pink; smallest opening angle of each Pex5 molecule on MDH) conformations of MP2. (Right panel) Overlay of distal and selected intermediate (violet; small and intermediate opening angle respectively for two Pex5 molecules on MDH) conformations of MP2. Arrows indicate the relative displacement of Pex5 density in the compared structures. The size of the arrow depicts the extent of displacement.

Supplementary Figure 10

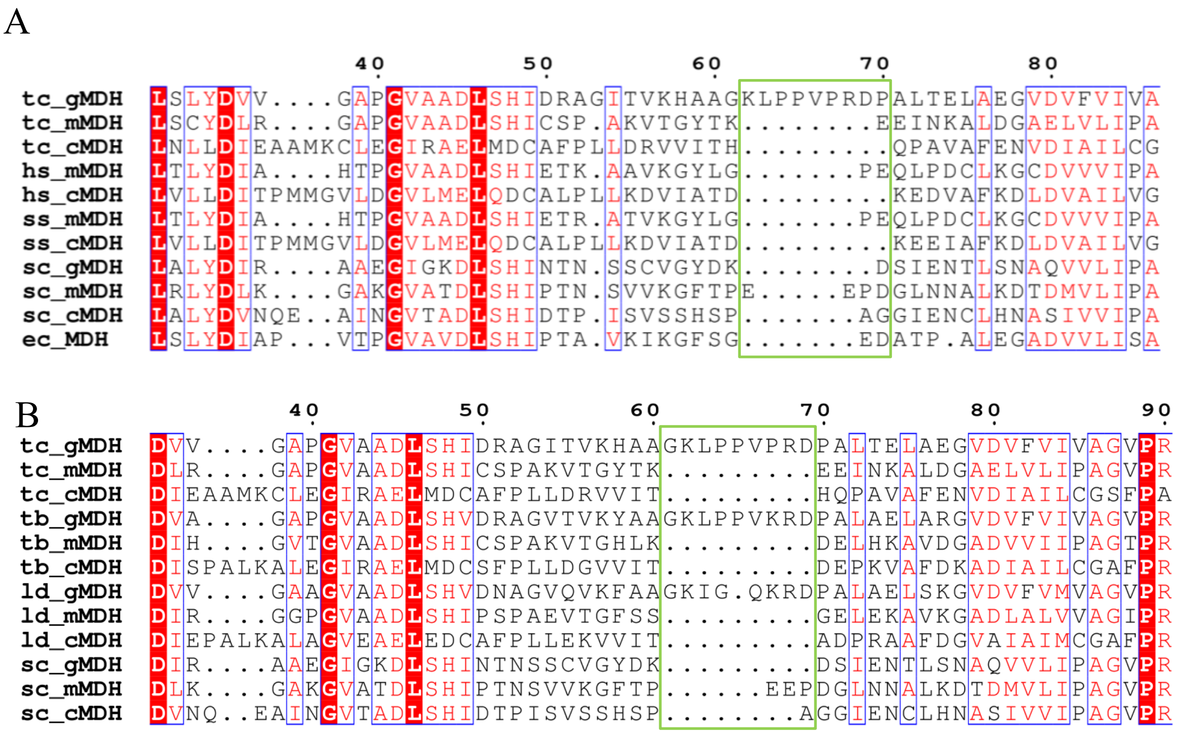

Supplementary Figure 10: Multiple sequence alignment of *T. cruzi* glycosomal MDH with subcellular MDH isoforms from different eukaryotes along with the *E. coli* MDH **(A)** and with organisms which compartmentalize glycolytic enzymes in the glycosomes **(B)** highlighting insertion of proline rich 9-mer (62-KLPPVPRDP-70) (green box) in glycosomal MDH. Acronyms: tc, *Trypanosoma cruzi*; hs, *Homo sapiens*; ss, *Sus scrofa*; sc, *Saccharomyces cerevisiae*; ec, *Escherichia coli*; tb, *Trypanosoma brucei*, ld, *Leishmania donovani*; gMDH, glycosomal MDH; cMDH, cytosolic MDH; mMDH, mitochondrial MDH. Conserved and partially conserved positions are highlighted by red background and blue boxes, respectively.

Supplementary Figure 11

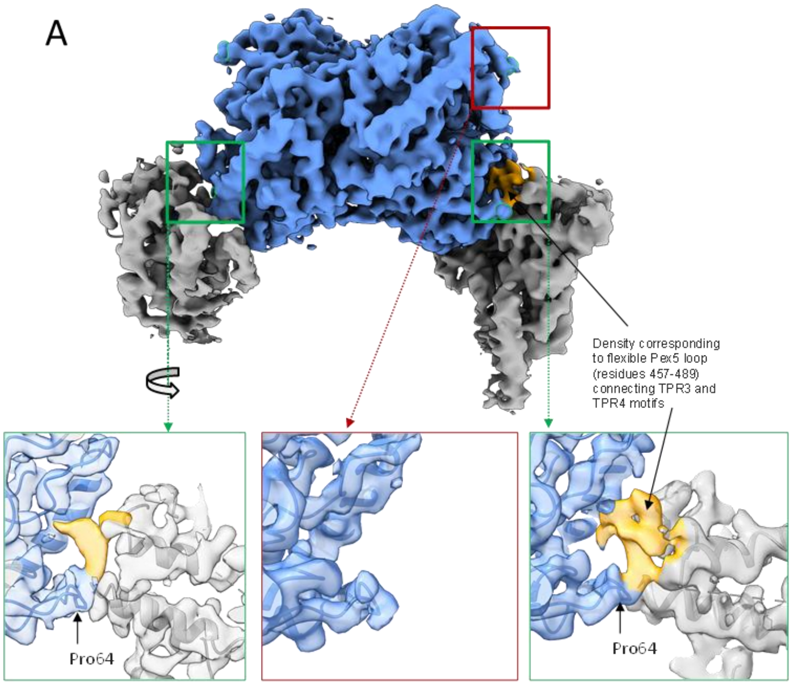

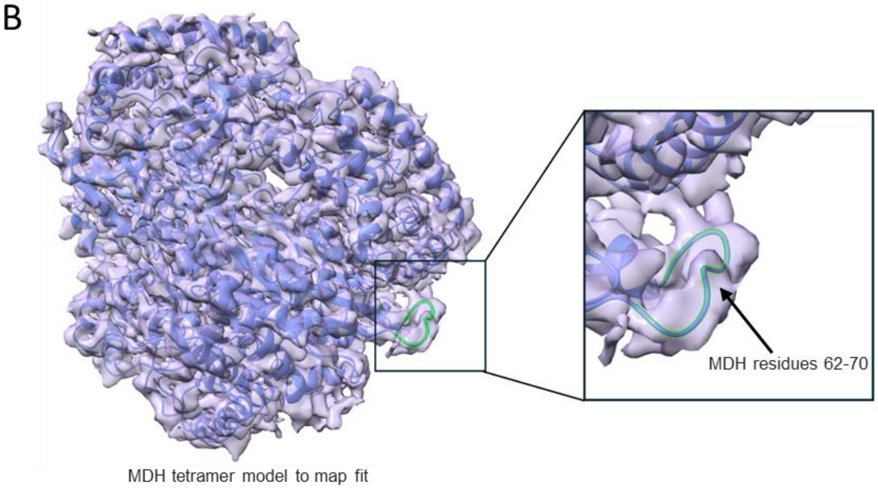

Supplementary Figure 11: Non-PTS1 interactions of Pex5 and MDH in MP2-d complex. **(A)** Density corresponding to the $Pex5_{457\text{-}489}$ loop (gold) transiently interacting with MDH (blue) in MP2-d map. The density is not seen in a groove close to the Pex5 unoccupied site (red box). See Figure 3 for analogous analysis of MP1-d map. **(B)** MDH model fit in cryo-EM map of MDH tetramer. The map was reconstructed by selecting particles containing MDH only in micrographs used for solution of a ternary complex. The map shows no density corresponding to the $Pex5_{457\text{-}489}$ loop near the MDH residues 62-70. The presence of the discussed density in MP-2 map and its absence in MDH map indicates that it originates form Pex5, while its positioning indicates it accounts for $Pex5_{457\text{-}489}$ loop.

Supplementary Figure 12

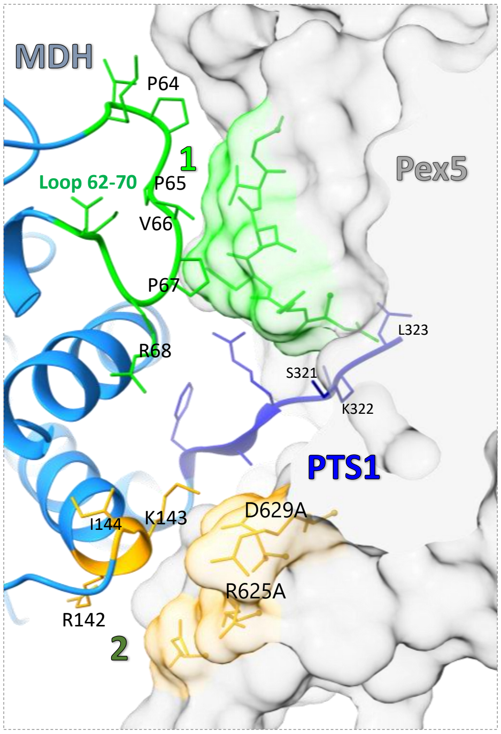

Supplementary Figure 12: PTS1 and non-PTS1 interactions of Pex5 and MDH in close state. Compare with right panel of main Figure 3A showing corresponding interactions in distal state.

Supplementary Figure 13

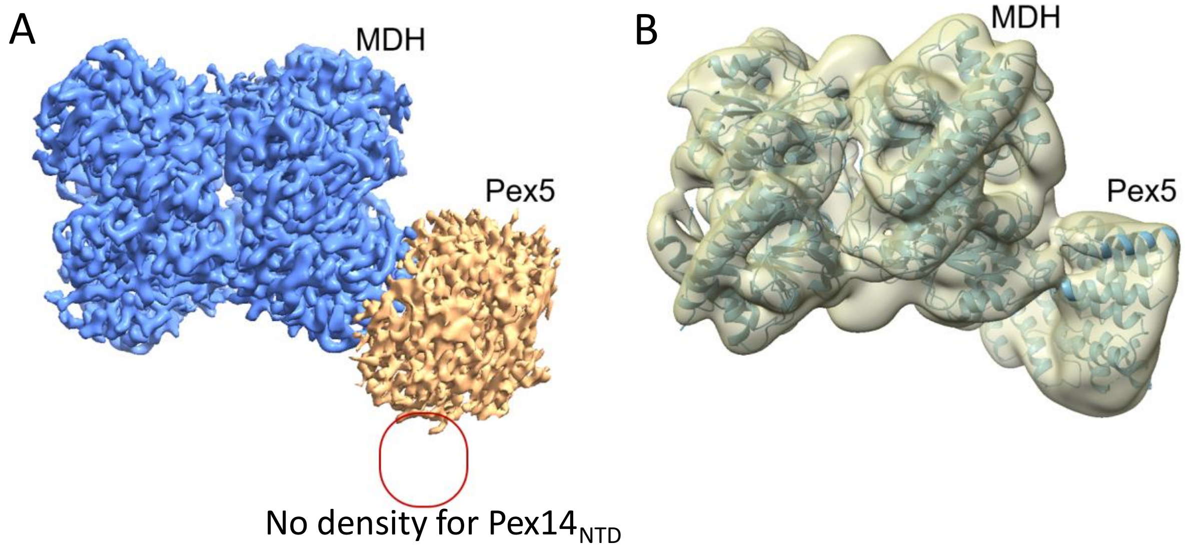

Supplementary Figure 13: Cryo-EM map of MDH-Pex5 binary complex obtained in the absence of $Pex14_{NTD}$. **(A)** High resolution cryo-EM map of MDH-Pex5 showing MDH density (blue) and Pex5 density (brown). Even though full length Pex5 was used for complex reconstitution the map shows density only for TPR domain indicating the unstructured, flexible nature of N-terminal domain of Pex5. No density is seen in the region indicated by red oval corresponding with the fact that complex was reconstituted in the absence of $Pex14_{NTD}$. **(B)** The MDH-Pex5 model fit in low pass filtered cryo-EM map again showing no density corresponding to $Pex14_{NTD}$ corresponding with the fact that $Pex14_{NTD}$ component was not used for complex reconstitution. The shown results indicate that the density present at the equivalent place in ternary complex corresponds to $Pex14_{NTD.}$

Supplementary Figure 14

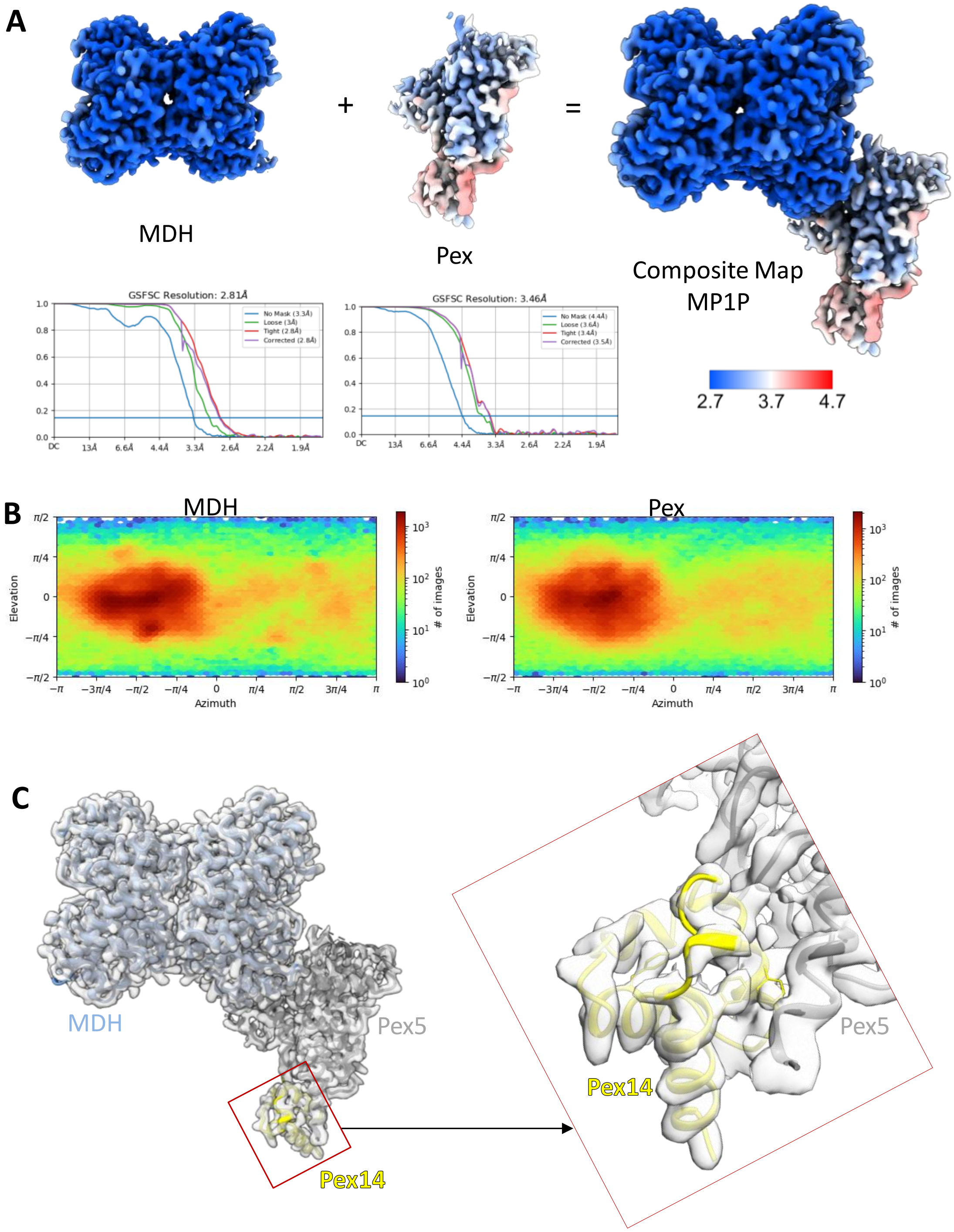

Supplementary Figure 14: Cryo-EM structure of *T. cruzi* MP1P complex. **(A)** Individual maps of MDH and Pex region are combined to generate the composite map of MP1P. The local resolution maps and global resolution map-to-map FSC curve (threshold 0.143) of individual maps are shown. **(B)** Angular distribution of particles, **(C)** Overall model-to-map fit with close-up view of Pex14 region.

Supplementary Figure 15

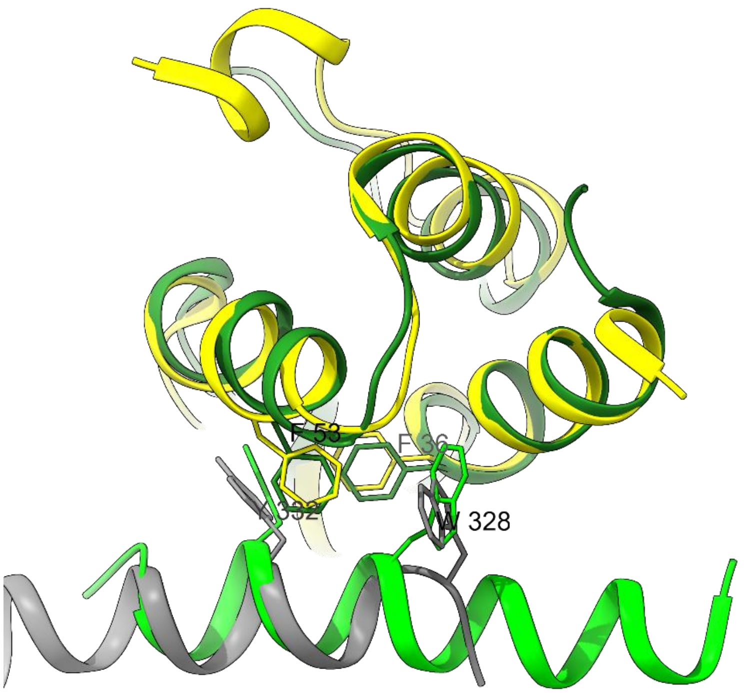

Supplementary Figure 15: Superimposed view of Pex5 Wxxx(F/Y) motif - Pex14$_{NTD}$ structure described by Neufeld et al[2] (PDB id: 2w84; light green - Pex5 Wxxx(F/Y) motif, dark green - Pex14$_{NTD}$) and corresponding Pex5 fragment and Pex14$_{NTD}$ of MP1P cryo-EM structure described in the present study (PDB id: 8gi0; Grey – Pex5 Wxxx(F/Y) motif; Yellow – Pex14$_{NTD}$). The RMSD between equivalent Cα (46) atoms of Pex14$_{NTD}$ and Pex5 Wxxx(F/Y) is ~1 Å indicating significant similarity between the two structures. Aromatic residues important for binding are highlighted in stick model.

Supplementary Figure 16

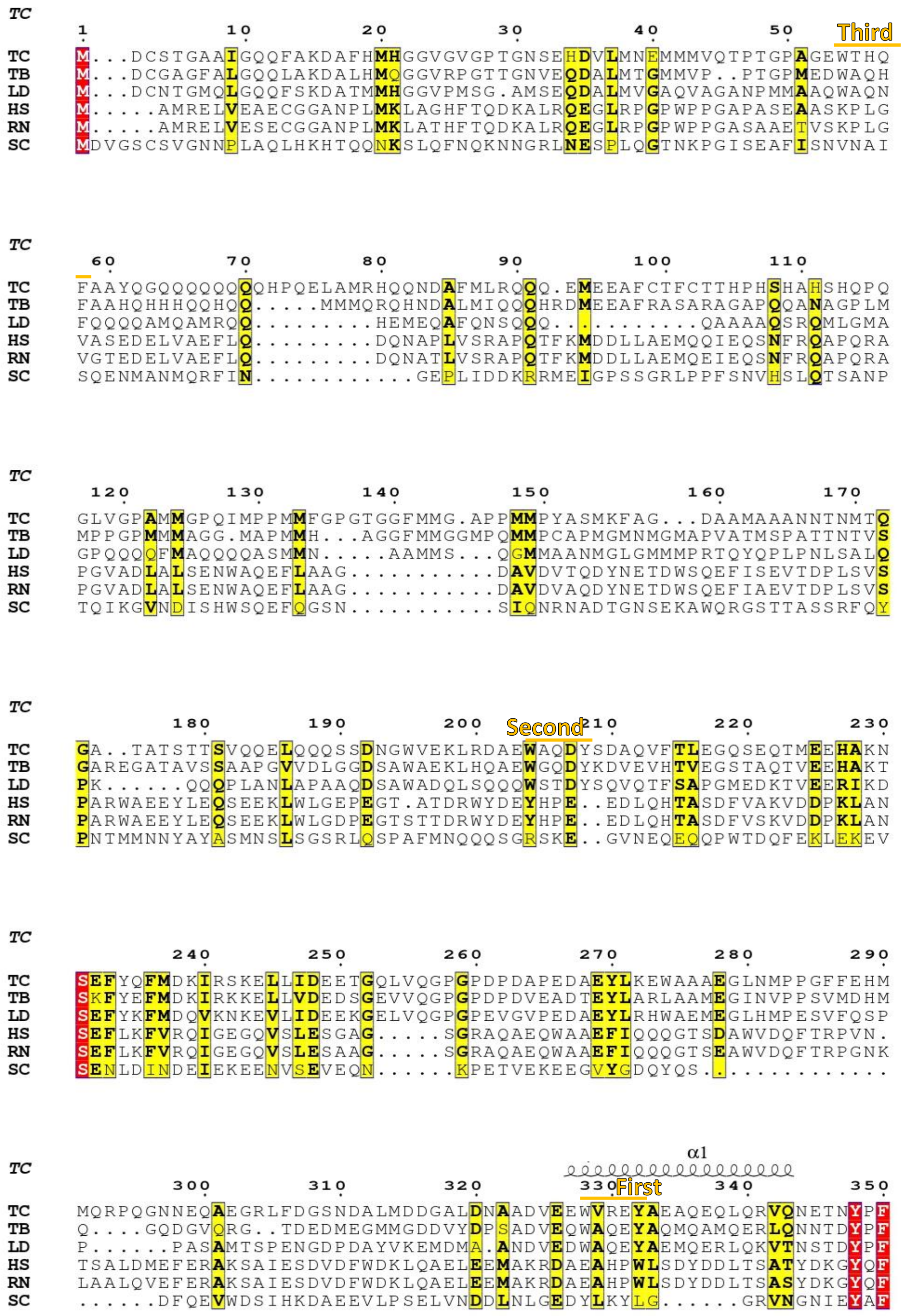

Supplementary Figure 16: Multiple sequence alignment of Pex5 homologues from *Trypanosoma cruzi* (TC), *Trypansosoma brucei* (TB), *Leishmania donovani* (LD), human (HS), *Rattus norvegicus* (RN) and *Saccharomyces cerevisiae* (SC). α#, α-helix number. Three Wxxx(F/Y) motifs in TC Pex5 are designated as First, Second and Third, respectively counting from C- to N-terminus. The residue conservation within 353-NNPYM-358 motif (non-Wxxx(F/Y) interaction site with $Pex14_{NTD}$) is indicated by blue box. TPR I to TPR VIII regions are indicated. Conserved and partially conserved positions are highlighted by red and yellow, respectively.

Supplementary Figure 16 (continue)

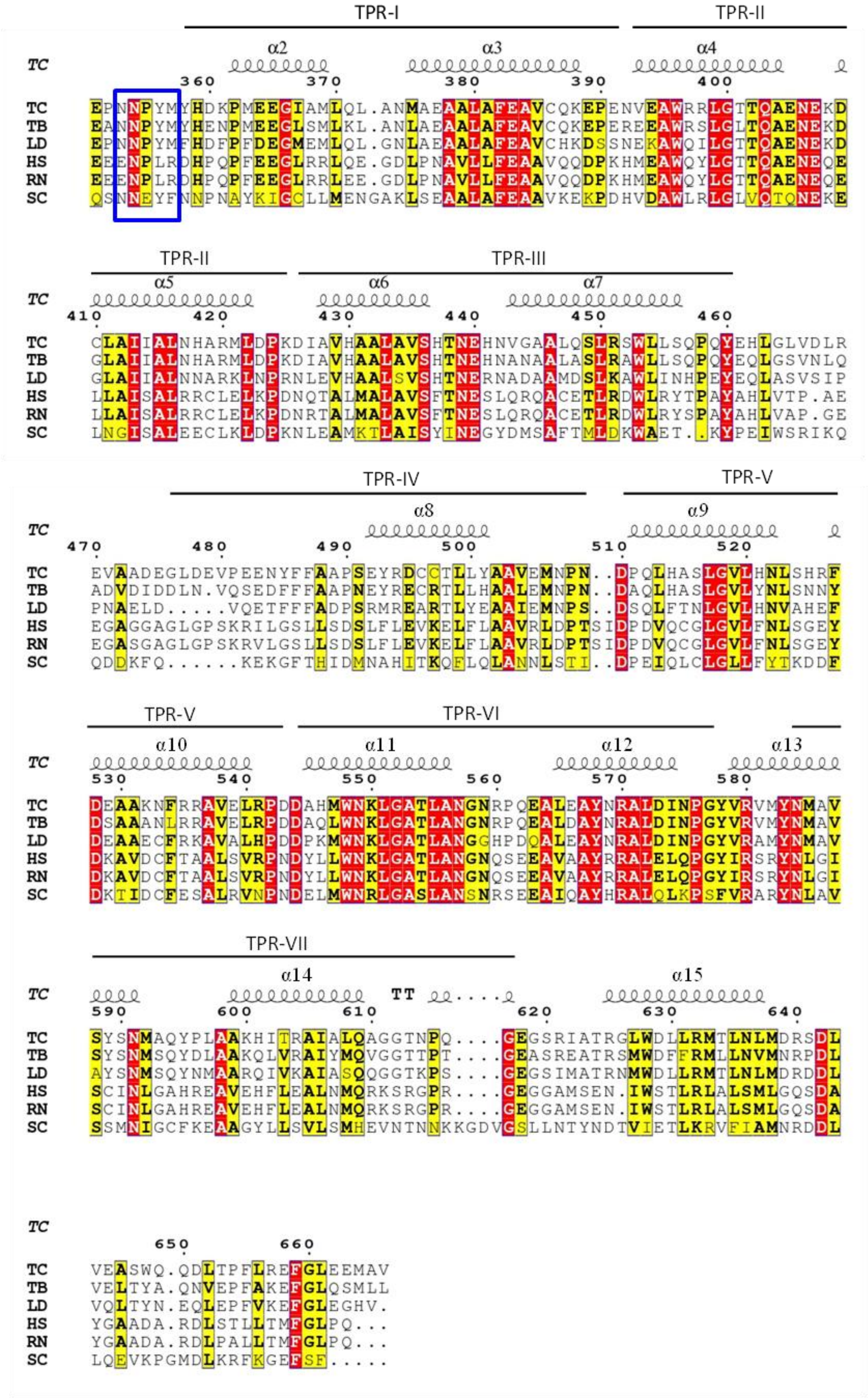

Supplementary Figure 17

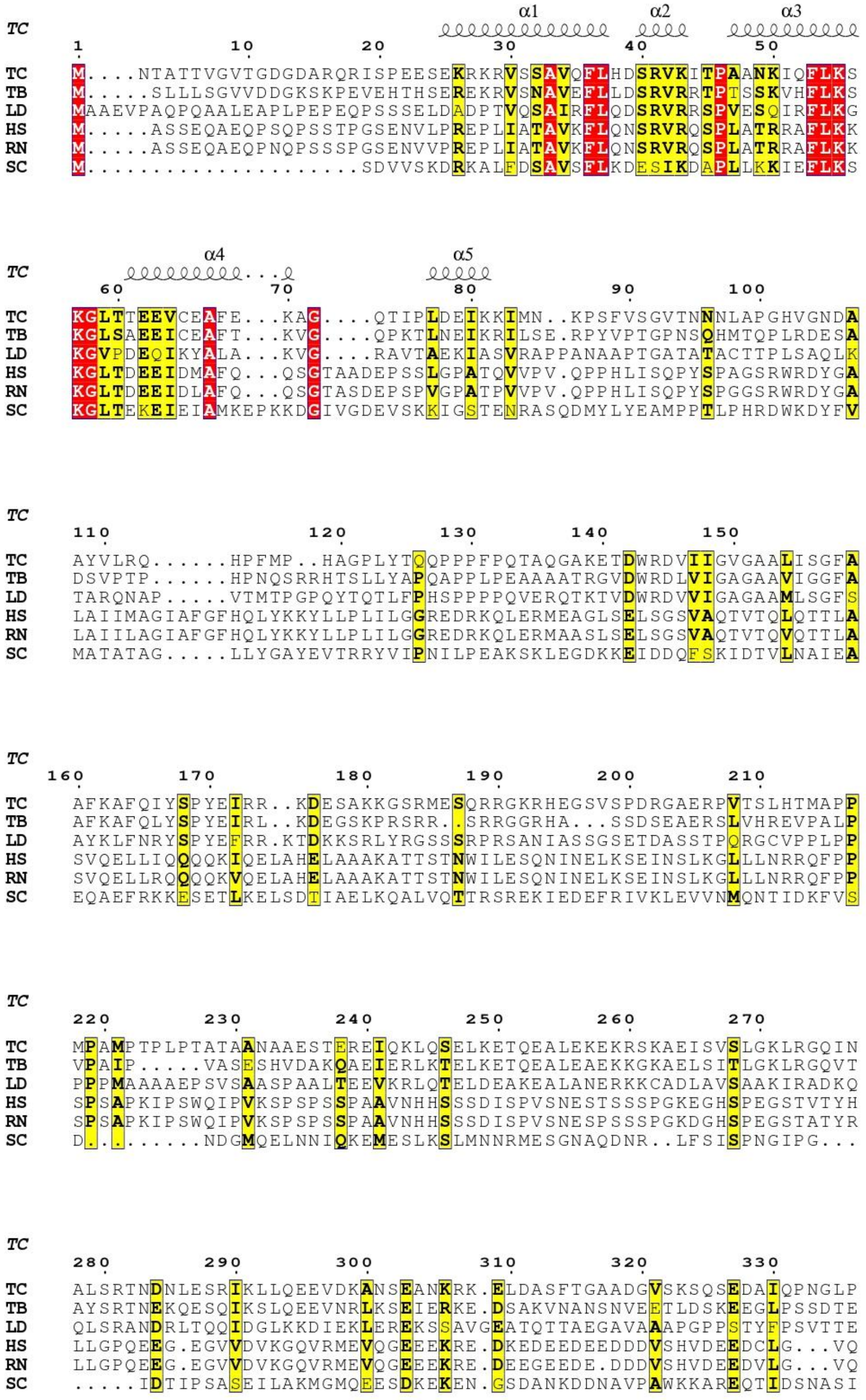

Supplementary Figure 17: Multiple sequence alignment of Pex14 homologues from *Trypanosoma cruzi* (TC), *Trypansosoma brucei* (TB), *Leishmania donovani* (LD), human (HS), *Rattus norvegicus* (RN) and *Saccharomyces cerevisiae* (SC). α#, α-helix number. Conserved and partially conserved positions are highlighted by red and yellow color, respectively.

Supplementary Figure 18:

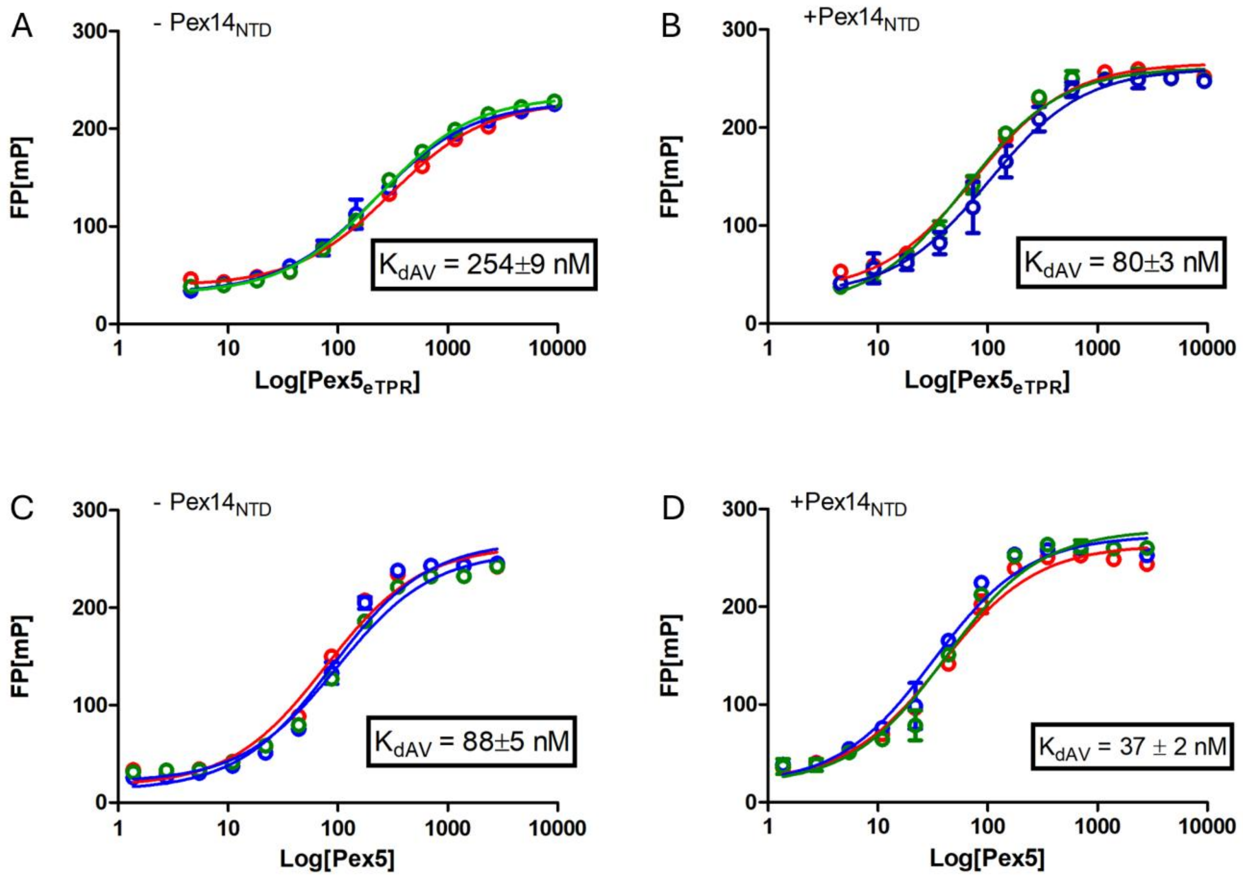

Supplementary Figure 18: Fluorescence Polarization based interaction assay. **(A, B)** Titration of PTS1 peptide with $Pex5_{eTPR}$ in **(A)** absence and **(B)** presence of excess $Pex14_{NTD}$. **(C, D)** Titration of PTS1 peptide with full length Pex5 in **(C)** absence and **(D)** presence of excess $Pex14_{NTD}$. **(all panels)** Each experiment was performed independently three times (red, green and blue), each point was determined in triplicate. Affinities (K_d) were determined by fitting**.** Average affinity (K_{dAV})is reported.

Supplementary Table 1: The details of cargo, Pex5 and Pex14 variants tested for the ternary complex reconstitution.

Cargo	Results	Pex5 variants	Pex14
• Malate dehydrogenase (MDH) (Uniprot: Q4DRD8)	MDH formed ternary complex which cryo-EM structure is described in present paper.	• Full length Pex5 (residues 1-666) (GenBank: PBJ69826.1)<br><br>• $Pex5_{eTPR}$ (extended TPR domain; residues 314-666) (GenBank: PBJ69826.1)	• $Pex14_{NTD}$ (residues 21-85) (GenBank: RNC55913.1)
• Phosphofructokinase (PFK) (GenBank: KAF5225211.1)	PFK formed ternary complex as evidenced by SEC, but never gave good 2D class averages in cryo-EM experiment.		
• Glyceraldehyde-3-phosphate dehydrogenase (GAPDH) (UniProt: P22513.1)	GAPDH formed ternary complex as evidenced by SEC but due to small size of the complex suffered from low signal-to-noise ratio in cryo-EM experiment. No satisfactory 2D class averages were obtained.		
• Phosphoglucose Isomerase (PGI) (PDB: 4QFH)	In our hands PGI did not interact with Pex5.		
• Glycerol Kinase (GK) (GenBank: ESS71147.1)	In our hands GK did not interact with Pex5. The observation was explained by steric occlusion of PTS1 signal in GK dimer as described in[3].		

Supplementary Table 2: Mutational analysis of $Pex14_{NTD}$-$Pex5_{eTPR}$ interface. The interaction of indicated mutants was characterized by Isothermal Titration Calorimetry (ITC) in the absence of PTS1 peptide. The p-value derived from the Welch's t-test (two-tailed) was used for comparing Kd values.

$Pex5_{eTPR}$	$Pex14_{NTD}$	Kd [nM]	ΔH [kJ/mol]	-TΔS [J/molK]	ΔG [kJ/mol]	N	Statistical significance (p-value)[1]
Wild type	Wild type	35 ± 5	-61.8 ± 0.4	19.3 ± 3.5	-42.6 ± 0.2	0.657 ± 0.002	nr
Wild type	Q52A	17 ± 4	-56.2 ± 0.6	11.8 ± 1.2	-44.4 ± 0.3	0.647 ± 0.003	<0.001
	A48F	59 ± 24	-73.7 ± 1.4	32.3 ± 6.2	-41.5 ± 0.6	0.680 ± 0.007	0.002
Y358A	Wild type	119 ± 35	-65.3 ± 1.4	25.6 ± 4.9	-39.7 ± 0.5	0.762 ± 0.01	<0.001
N353E		48 ± 11	-61.9 ± 0.7	20 ± 8.9	-41.8 ± 0.6	0.738 ± 0.005	<0.001
GSSG		122 ± 17	-59.4 ± 0.7	19.9 ± 0.6	-39.5 ± 0.6	0.568 ± 0.004	<0.001

[1] Welch's t-test (two-tailed) p-value for comparing Kd values. Variants are compared to wild type.

Supplementary Table 3: Statistics for cryo-EM data collection, image processing and model building

**Data collection and processing**	**MP1-c**	**MP1-d**	**MP2-c**	**MP2-d**	**MP1P**	**MDH alone**	**MDH-PEX5**
**PDB entry**	8GGD	8GGH	8GH2	8GH3	8GI0	9FEE	9FEF
**EMBD entry**	40003	40008	40031	40032	40056	50339	50340
**Voltage (kV)**	300	300	300	300	300	300	300
**Magnification**	105k	105k	105k	105k	105k	105k	105k
**Electron exposure (e$^-$/ Å^2)**	42.25	42.25	42.25	42.25	42.25	42.25	42.79
**Defocus range (µm)**	-0.9 to -3.0	-0.9 to -3.0	-0.9 to -3.0	-0.9 to -3.0	-0.9 to -3.0	-0.9 to -3.0	-0.9 to - 3.38
**Pixel size (Å/pixel)**	0.86	0.86	0.86	0.86	0.86	0.86	0.84
**Symmetry imposed**	C1	C1	C1	C1	C1	D2	C1
**Particle images (no.)**	25,845	17,940	12,276	12,554	482,240	312,103	538,505
**Map global resolution (Å)**	3.33	3.29	3.66	3.53	2.8/3.5	3.03	2.98
**Local resolution FSC threshold**	0.143	0.143	0.143	0.143	0.143	0.143	0.143
**Refinement**							
**Map sharpening (Å^2)**	40	35	20	20	Sharpened by EMReady	117.1	80.9
Model composition							
**No. of proteins chain (MDH/Pex5/Pex14$_{NTD}$)**	4/1/0	4/1/0	4/2/0	4/2/0	4/1/1	4/0/0	4/1/0
**Non-hydrogen atoms**	11,724	11,798	13,941	13,519	12,191	9,112	11,192
**Residues**	1,567	1,577	1,846	1,798	1,625	1,236	1,502
Validation							
**MolProbity score**	2.30	1.93	2.08	1.96	1.39	1.73	1.75
**Clash score**	8.47	6.89	9.08	9.60	3.56	4.99	6.46
**Rotamer outliers (%)**	2.13	0.00	0.00	0.00	0.68	0.1	0.33
R.M.D deviations							
**Bond Length (Å)**	0.004	0.003	0.003	0.002	0.004	0.004	0.008
**Bond Angle (°)**	0.724	0.640	0.706	0.672	0.687	0.582	0.796
Ramachandran plot (%)							
**Outliers**	1.03	0.58	0.55	0.11	0.00	0.00	0.00
**Allowed**	10.80	9.39	11.16	7.13	3.68	7.46	5.89
**Favoured**	88.17	90.03	88.29	92.76	96.32	92.54	94.11
**Rama-Z-score**	-1.26	-0.95	-1.01	-0.92	-0.51	-1.4	0.13
Model vs. data							
**CC (mask)**	0.83	0.85	0.81	0.80	0.83	0.87	0.87
**CC (volume)**	0.82	0.84	0.79	0.78	0.84	0.85	0.81

Supplementary Table 4: Details of protein constructs used in this study

Protein name	Construct Range (aa numbers)	Uniprot entry (according to which the aa are numbered in prior column)	Experiments for which the construct was used	Mutants and details
Pex5	1-666	V5B7T1	Cryo-EM, FP	-
$Pex5_{eTPR}$ (truncated Pex5 construct slightly exceeding the region defined by density in MP1P structure [residues 327-462 and 487-653]; the construct encompasses extended TPR domain including upstream helix containing the Wxxx(F/Y) motif)	314-666	V5B7T1	ITC, FP	$Pex5_{eTPR}$(R625A, D629A)
				$Pex5_{eTPR}$(P490R)
				$Pex5_{eTPR}$(Δ470-480)
				$Pex5_{eTPR}$(Y358A)
				$Pex5_{eTPR}$(N353E)
				$Pex5_{eTPR}$(GSGS) GSGS linker was added between Gln338 and Leu339 to separate TPR domain and Wxxx(Y/F) containing helix
MDH	1-323	Q4DRD8	ITC, Cryo-EM	MDH(GSGS): GSGS linker was added between Phe318 and Ala319
				MDH(Δ62-70) Residues 62-70 substituted with GS

$Pex14_{NTD}$	21-85	Q4D1H5	ITC, FP, Cryo-EM	$Pex14_{NTD}$(Q52A)
				$Pex14_{NTD}$(A48F)

25

SUPPLEMENTARY METHODS

Expression and purification of individual proteins

Full length Pex5

Escherichia coli BL21 (DE3) was transformed with N-terminally 6x His-tagged Pex5 gene containing pET24(+) vector and grown in Luria-Bertani (LB) medium at 37 °C until the optical density (OD_{660}) of the culture reached between 0.6 and 0.8. The culture was allowed to cool down to 25°C, and the expression of the Pex5 protein was induced by adding 0.5 mM isopropyl β-d-1-thiogalactopyranoside (IPTG). Expression was continued for ~18 hours with 200 RPM shaking. The cell mass was harvested by centrifugation and re-suspended in lysis buffer (50 mM Tris-HCl, 300 mM NaCl, 20 mM Imidazole, pH 8.0, 5mM 2-Mercaptoethanol). Commercially available protease inhibitor cocktail and DNAse I were added to the cell suspension prior to the cell lysis. Cells were lysed by the probe sonicator while being in the ice-bucket. The temperature threshold was set to 16°C. Cell debris was removed by centrifugation. Cell lysate (supernatant after centrifugation) was loaded to the glass column packed with Ni-NTA (HisPur™ Ni-NTA Resin, Thermo Fisher Scientific) to capture the his-tagged Pex5 protein. The protein bound resin was washed with 5 column volume (CV) of the lysis buffer and the protein of interest was eluted by 2 CV of the elution buffer (50 mM Tris-HCl, 300 mM NaCl, 300 mM Imidazole, pH 8.0, 5mM 2-Mercaptoethanol). Eluted protein sample was concentrated by ultra filtration (Amicon® Ultra Centrifugal Filter, Millipore Sigma) and further purified by passing through S200 gel filtration column in running buffer (20 mM HEPES buffer, 150 mM NaCl, pH 8.0, 2mM 2-Mercaptoethanol). Eluted protein was concentrated and plunge frozen in liquid nitrogen for storage at -80°C until further use. The concentration of protein was determined using Nano-drop spectrophotometer. 1.00 OD = 1.00 mg/mL was considered for Pex5 concentration derivation as per its absorbance coefficient.

Malate dehydrogenase (MDH)

Escherichia coli BL21 (DE3), transformed with N-terminally 6x His-tagged MDH gene containing pET24(+) vector, were grown in Luria-Bertani (LB) medium at 37 °C until the optical density (OD660) of media reached between 0.6 and 0.8. The grown culture was induced for MDH expression by adding 0.5 mM IPTG and the culture was continued for ~5 hours with 200 RPM shaking. MDH purification was conducted using a similar protocol to full length Pex5 except the 10kDa MWCO device (Amicon® Ultra Centrifugal Filter, 10 kDa MWCO, Millipore Sigma) was used for concentrating the protein. The concentration of protein was measured using Nano-drop spectrophotometer. 0.25 OD = 1.00 mg/mL was considered for MDH concentration derivation as per its absorbance coefficient.

$Pex14_{NTD}$

Escherichia coli BL21 (DE3), transformed with N-terminally 6x His-tagged $Pex14_{NTD}$ (residues 21-85) gene containing pET24(+) vector, were grown in Luria-Bertani (LB) medium at 37 °C until the optical density (OD660) of media reached between 0.6 and 0.8. The grown culture was cooled down to 4°C, and the expression of the Pex14 protein was induced by adding 0.5 mM isopropyl β-d-1-thiogalactopyranoside (IPTG) and the culture was continued for ~18 hours with 200 RPM shaking. $Pex14_{NTD}$ purification was conducted using a similar protocol to full length Pex5 with few modifications. The 6X His-tag was cleaved

off by after Ni-NTA step of purification. The additional step of reverse Ni-NTA was performed to remove the cleaved 6x His-tag. The 3kDa MWCO device (Amicon® Ultra Centrifugal Filter, Millipore Sigma) was used for concentrating the $Pex14_{NTD}$ and S75 column was used for the last step of purification. The concentration of protein was measured using Nano-drop spectrophotometer. 0.1 OD = 1.00 mg/mL was considered for $Pex14_{NTD}$ concentration derivation as per its absorbance coefficient.

*Truncated Pex5 (*residues 314-666; $Pex5_{eTPR}$*)*

Escherichia coli BL21 (DE3), transformed with N-terminally 6x His-tagged truncated gene of $Pex5_{eTPR}$ (corresponding to residues 314-666) containing pET24(+) vector, were grown in Luria-Bertani (LB) medium at 37 °C until the optical density (OD660) of media reached between 0.6 and 0.8. The grown culture was cooled down to 16°C, and the expression of the $Pex5_{eTPR}$ protein was induced by adding 0.5 mM isopropyl β-d-1-thiogalactopyranoside (IPTG) and the culture was continued for ~18 hours with 200 RPM shaking. $Pex5_{eTPR}$ purification was conducted using a similar protocol to full length Pex5 except the 10kDa MWCO device (Amicon® Ultra Centrifugal Filter, Millipore Sigma) was used for concentrating the protein and S75 column was used for the last step of purification. The concentration of protein was measured using Nano-drop spectrophotometer. 1.00 OD = 1.00 mg/mL was considered for $Pex5_{eTPR}$ concentration derivation as per its absorbance coefficient.

References

1. Goddard, T.D. et al. UCSF ChimeraX: Meeting modern challenges in visualization and analysis. *Protein Science: A Publication of the Protein Society* **27**, 14-25 (2018).
2. Neufeld, C. et al. Structural basis for competitive interactions of Pex14 with the import receptors Pex5 and Pex19. *The EMBO Journal* **28**, 745-754 (2009).
3. Lipiński, O., Sonani, R.R. & Dubin, G. Crystal structure of glycerol kinase from Trypanosoma cruzi, a potential molecular target in Chagas disease. *Biological Crystallography Section D* **80**, 629-638 (2024).
4. Punjani, A. & Fleet, D.J. 3D variability analysis: Resolving continuous flexibility and discrete heterogeneity from single particle cryo-EM. *Journal of structural biology* **213**, 107702 (2021).
5. Pettersen, E.F. et al. UCSF Chimera—a visualization system for exploratory research and analysis. *Journal of computational chemistry* **25**, 1605-1612 (2004).
