# Supplementary material for: Structure of Trypanosoma peroxisomal import complex unveils conformational heterogeneity
Source: Nat Commun. 2025 Dec 11;16:11398. doi: 10.1038/s41467-025-66207-8 (PMC12738837; doi:10.1038/s41467-025-66207-8)
Supplement: Supplementary file 2 — Description of Additional Supplementary Files [file 41467_2025_66207_MOESM2_ESM.pdf]

Description of Additional Supplementary Files

File Name: Supplementary Movie 1
Description: Results of 3D variability analysis (3DVA) by cryoSPARC showing the conformational variability of Pex5 relative to the MDH in MP1 complex. The movie is created in UCSF Chimera using the density map series of MP1 generated by cryoSPARC 3DVA.

File Name: Supplementary Movie 2
Description: Results of 3D variability analysis (3DVA) by cryoSPARC showing the conformational variability of Pex5 relative to the MDH in MP2 complex. The movie is created in UCSF Chimera using the density map series of MP2 generated by cryoSPARC 3DVA.

File Name: Supplementary Movie 3
Description: Morphing between the close and distal conformations of MP1 complex - overview of the complex. The color scheme is the same as in Figure 2. The movie is created in UCSF Chimera.

File Name: Supplementary Movie 4
Description: Morphing between the close and distal conformations of MP1 complex - the view from Pex5 side. The color scheme is the same as in Figure 2. The movie is created in UCSF Chimera.

File Name: Supplementary Movie 5
Description: Morphing between the close and distal conformations of MP2 complex - overview of the complex. The color scheme is the same as in Figure 2. The movie is created in UCSF Chimera.

File Name: Supplementary Movie 6
Description: Morphing between the close and distal conformations of MP2 complex - the view from right hand side Pex5 (as a hand reference please see orientation in Supplementary Figure 5 and Supplementary Figure 6). The color scheme is the same as in Figure 2. The movie is created in UCSF Chimera.

File Name: Supplementary Movie 7
Description: Morphing between the close and distal conformations of MP2 complex - the view from left hand side Pex5 (as a hand reference please see orientation shown in Supplementary Figure 5 and Supplementary Figure 6). The color scheme is the same as in Figure 2. The movie is created in UCSF Chimera.

File Name: Supplementary Movie 8
Description: The twisting of Pex5 relative to the axis roughly parallel to TPR3 motif. The color scheme is the same as in Figure 2. The movie is created in UCSF Chimera.
