# Supplementary material for: Structure of Trypanosoma peroxisomal import complex unveils conformational heterogeneity
Source: Nat Commun. 2025 Dec 11;16:11398. doi: 10.1038/s41467-025-66207-8 (PMC12738837; doi:10.1038/s41467-025-66207-8)
Supplement: Supplementary file 11 — Reporting Summary [file 41467_2025_66207_MOESM11_ESM.pdf]

nature portfolio

Corresponding author(s): Grzegorz Dubin
Last updated by author(s): Aug 22, 2025

Reporting Summary

Nature Portfolio wishes to improve the reproducibility of the work that we publish. This form provides structure for consistency and transparency in reporting. For further information on Nature Portfolio policies, see our Editorial Policies and the Editorial Policy Checklist.

Statistics

For all statistical analyses, confirm that the following items are present in the figure legend, table legend, main text, or Methods section.

n/a	Confirmed	
☐	☒	The exact sample size (*n*) for each experimental group/condition, given as a discrete number and unit of measurement
☒	☐	A statement on whether measurements were taken from distinct samples or whether the same sample was measured repeatedly
☐	☒	The statistical test(s) used AND whether they are one- or two-sided<br>*Only common tests should be described solely by name; describe more complex techniques in the Methods section.*
☒	☐	A description of all covariates tested
☒	☐	A description of any assumptions or corrections, such as tests of normality and adjustment for multiple comparisons
☒	☐	A full description of the statistical parameters including central tendency (e.g. means) or other basic estimates (e.g. regression coefficient) AND variation (e.g. standard deviation) or associated estimates of uncertainty (e.g. confidence intervals)
☐	☒	For null hypothesis testing, the test statistic (e.g. F, t, r) with confidence intervals, effect sizes, degrees of freedom and P value noted<br>*Give P values as exact values whenever suitable.*
☒	☐	For Bayesian analysis, information on the choice of priors and Markov chain Monte Carlo settings
☒	☐	For hierarchical and complex designs, identification of the appropriate level for tests and full reporting of outcomes
☒	☐	Estimates of effect sizes (e.g. Cohen's *d*, Pearson's *r*), indicating how they were calculated

Our web collection on statistics for biologists contains articles on many of the points above.

Software and code

Policy information about availability of computer code

Data collection	EPU
Data analysis	cryoSPARC v3.3, cryoSPARC v4.5.1, UCSF ChimeraX v1.7.1, PyMol v3.1.0

For manuscripts utilizing custom algorithms or software that are central to the research but not yet described in published literature, software must be made available to editors and reviewers. We strongly encourage code deposition in a community repository (e.g. GitHub). See the Nature Portfolio guidelines for submitting code & software for further information.

Data

Policy information about availability of data

All manuscripts must include a data availability statement. This statement should provide the following information, where applicable:

- Accession codes, unique identifiers, or web links for publicly available datasets
- A description of any restrictions on data availability
- For clinical datasets or third party data, please ensure that the statement adheres to our policy

Density maps of complexes, MP1P, MP1-c, MP1-d, MP2-c, MP2-d, MDH-Pex5 and MDH alone are available at the electron microscopy database (EMBD) with accession ids, EMD-40056, EMD-40003, EMD-40008, EMD-40031, EMD-40032, EMD-50340 and EMD-50339 respectively. Refined 3D-structure coordinates of complexes, MP1P, MP1-c, MP1-d, MP2-c, MP2-d, MDH-Pex5 and MDH alone are available at the protein data bank (PDB) with accession ids, 8GI0, 8GGD, 8GGH, 8GH2, 8GH3, 9FEF and 9FEE and, respectively.

Research involving human participants, their data, or biological material

Policy information about studies with human participants or human data. See also policy information about sex, gender (identity/presentation), and sexual orientation and race, ethnicity and racism.

Reporting on sex and gender	N/A
Reporting on race, ethnicity, or other socially relevant groupings	N/A
Population characteristics	N/A
Recruitment	N/A
Ethics oversight	N/A

Note that full information on the approval of the study protocol must also be provided in the manuscript.

Field-specific reporting

Please select the one below that is the best fit for your research. If you are not sure, read the appropriate sections before making your selection.

[x] Life sciences [ ] Behavioural & social sciences [ ] Ecological, evolutionary & environmental sciences

For a reference copy of the document with all sections, see nature.com/documents/nr-reporting-summary-flat.pdf

Life sciences study design

All studies must disclose on these points even when the disclosure is negative.

Sample size	No statistical methods were used to predetermine sample size. The number of micrographs and particle images collected was based on standard practices in single-particle cryo-EM to achieve the desired resolution. The final dataset included 8169 micrographs and different numbers of particles images for each structure, which yielded a reconstruction in the range of 3.3-3.7 Å.
Data exclusions	Micrographs were excluded based on established quality criteria, including excessive drift, poor contrast, contamination, or defocus outside the desired range. Particle images were excluded during 2D classification steps based on heterogeneity, alignment quality, or failure to converge to a consistent class. All exclusions were made using automated and semi-automated methods in cryoSPARC.
Replication	Multiple rounds of 2D and 3D classification were used to confirm the reproducibility of structural features. Biological replicates were not applicable in this structural determination study, but reconstruction was consistent across independently processed subsets of the data.
Randomization	Randomization was not applicable to this study, as it involved in vitro structural analysis of purified macromolecules using cryo-EM.
Blinding	Blinding was not applicable, as data processing and interpretation were based on objective computational methods and did not involve treatment groups or subjective assessments.

Reporting for specific materials, systems and methods

We require information from authors about some types of materials, experimental systems and methods used in many studies. Here, indicate whether each material, system or method listed is relevant to your study. If you are not sure if a list item applies to your research, read the appropriate section before selecting a response.

Materials & experimental systems

n/a	Involved in the study	
[x]	[ ]	Antibodies
[x]	[ ]	Eukaryotic cell lines
[x]	[ ]	Palaeontology and archaeology
[x]	[ ]	Animals and other organisms
[x]	[ ]	Clinical data
[x]	[ ]	Dual use research of concern
[x]	[ ]	Plants

Methods

n/a	Involved in the study	
[x]	[ ]	ChIP-seq
[x]	[ ]	Flow cytometry
[x]	[ ]	MRI-based neuroimaging

Plants

Seed stocks	N/A
Novel plant genotypes	N/A
Authentication	N/A
